# A community-based peer-support group intervention “Paths to EvERyday life” (PEER) added to service as usual for adults with vulnerability to mental health difficulties – a study protocol for a randomized controlled trial

**DOI:** 10.1186/s13063-022-06670-6

**Published:** 2022-09-02

**Authors:** Chalotte Heinsvig Poulsen, Cecilie Høgh Egmose, Bea Kolbe Ebersbach, Carsten Hjorthøj, Lene Falgaard Eplov

**Affiliations:** 1 Copenhagen Research Center for Mental Health (CORE), Mental Health Center Copenhagen, University of Copenhagen, Copenhagen, Denmark; 2 Department of Public Health, Section of Epidemiology, University of Copenhagen, Copenhagen, Denmark

**Keywords:** Peer support, Mental vulnerability, Mental health difficulties, Volunteer peers, Personal recovery, Community-based, Randomized controlled trial

## Abstract

**Background:**

The number of people struggling with vulnerability to mental health difficulties is increasing worldwide, and there is a need for new interventions, to prevent more people from developing serious mental illnesses. In recent years, peer support has been suggested as a key element in creating person-centered interventions in mental health services. However, the evidence for peer support is not yet established. We aim to investigate the effect of a 10-week peer-support intervention “Paths to EvERyday life” (PEER) added to service as usual (SAU) versus SAU alone in a Danish municipality setting.

**Methods:**

A two-armed, investigator-initiated, multi-municipal, parallel-group superiority trial to investigate the effectiveness of the PEER intervention added to SAU compared to SAU alone. A total of 284 participants will be recruited from the municipal social services in the participating municipalities and by self-referrals and randomly assigned to (1) the PEER intervention added to SAU or (2) SAU. The primary outcome is a self-assessed personal recovery (Questionnaire about the process of recovery (QPR-15)) at end of the intervention. The secondary outcomes are self-assessed empowerment (Empowerment Scale Rogers (ESR)), quality of life (The Manchester Short Assessment of Quality of life (MANSA)), and functioning (Work and Social Adjustment Scale (WSAS)).

**Discussion:**

This trial will test a new community-based peer-support intervention, and if the intervention proves to be effective, the goal is that future integration of this intervention will improve individual recovery and mental health and reduce the societal burden of individuals seeking municipal social support and/or mental health services.

**Trial registration:**

ClinicalTrials.gov NCT04639167. Registered on Nov. 19, 2020.

## Administrative information

Note: the numbers in curly brackets in this protocol refer to SPIRIT checklist item numbers. The order of the items has been modified to group similar items.

**Table:** 

Title {1}	An early community-based peer-support intervention “Paths to EvERyday life” (PEER) added to service as usual for adult people with vulnerability to mental health difficulties – a study protocol for a randomized controlled trial
Trial registration {2a and 2b}.	ClinicalTrials.gov, NCT04639167; registered on Nov. 19, 2020
Protocol version {3}	Version 4.0, 19.03.2021
Funding {4}	VELUX FOUNDATION, Tobaksvejen 10, 2860 Søborg, DK
Author details {5a}	CHP drafted the manuscript. CHP and CHE conducted the evaluation of the pilot study and participated in revision of the manual, planning the intervention and designing the study, and read and critically revised the manuscript. BKE participated in planning and designing the interventions and read and critically revised the manuscript. CH participated in the statistical analysis plan and critically revised the manuscript. LFE developed the trial, participated in planning and designing the intervention and the study design, and read and critically revised the manuscript. All authors read, improved, and approved the final manuscript
Name and contact information for the trial sponsor {5b}	DMSc, PhD Lene Falgaard Eplov (Principal investigator) Copenhagen Research Center for Mental Health (CORE), Recovery & Inclusion, Mental Health Center Copenhagen Gentofte Hospitals Vej 15, Entrance 3A, 4^th^ floor 2900 Hellerup, DK, Email: lene.falgaard.eplov@regionh.dk Phone: 0045 38647461
Role of sponsor {5c}	Study sponsor developed the trial, participated in planning and designing the intervention and the study design. Study sponsor will have ultimate authority over data collection, analysis and interpretation of data; writing of the report and articles; and the decision to submit the report and articles for publication. The Peer Partnership association is a Non-governmental Organization (NGO), which is housed in The Social Network association in Copenhagen, manages the operational part of the trial i.e. the collaboration with the local coordinators in each municipality, the training and supervision of the volunteer peers delivering the intervention. The steering group of the project keep track of the progress and in decisions regarding the scientific content of the project. The study was sponsored by The VELUX FOUNDATION. The Peer Partnership association, The Social Network association, the steering group and the VELUX FOUNDATION will not take part in decisions regarding data analysis, nor the interpretation or the publication of results.

## Introduction

### Background and rationale {6a}

Today, peer support and peer support interventions are widely used and are also finding their way into the established mental health services [1]. Peer support has been declared to be the best practice for supporting individual recovery by the American federal health program Medicare & Medicaid service [2]. ImROC (Implementing Recovery through Organizational Change), a large English organization, that works closely with the National Health Services, also states that peer support probably is the single most important factor for changing services to become more recovery-oriented [3]. However, the evidence lags after the use, as reviews show that the evidence for peer support is not yet established. The effect of peer support has been studied in randomized controlled trials (RCTs), in qualitative studies, and in a range of reviews [1, 4–10]. In general, earlier reviews found very few RCTs that were well performed and of high quality. The most recent systematic reviews with meta-analysis, two Cochrane reviews from 2013 [9] and 2019 [10], as well as two reviews from 2014 [5, 6] found that there was little or no effect of peer support on clinical recovery, i.e., symptom reduction and improvement in functioning [5, 6, 9], as well as no effect on hospital admission or all-cause death [10]. The reviews did find indications that peer support has small positive effects on outcome measures related to personal recovery such as hope and empowerment [5, 6, 10]. However, these data are mostly derived from single small trials and currently, the data are insufficient to draw any firm conclusions. More RCT studies are needed due to the low quality of most of the included studies in these reviews.

In Denmark, individuals experiencing vulnerability to mental health difficulties—defined in this project as individuals diagnosed with a mental illness and/or who are affected by mental health dissatisfaction to a degree that limits the unfolding of their life—can get different individual- or group-based offers in the municipal social service depending on the individual municipality. In this study protocol, we primarily aimed to investigate the effect of a peer-support intervention consisting of a voluntary peer-led 10-week group course “Paths to EvERyday life” (PEER) added to service as usual (SAU) in a Danish municipality setting—compared to SAU, in a superiority randomized two-armed trial. We hypothesize, that individuals with vulnerability to mental health difficulties who choose to participate in the PEER intervention, i.e., the 10-week group course delivered by two peers in addition to SAU, gain a significantly increased experience of self-assessed personal recovery compared to individuals who receive SAU only, measured at baseline and at post-intervention.

### Objectives {7}

The primary aim of the PEER trial is to compare the effect on the self-assessed personal recovery of the following interventions: (1) PEER intervention added to SAU and (2) SAU. An additional component of the PEER intervention is that the participants have if needed an opportunity for individual companionship to e.g., the group course, local communities, volunteer work, education, and mental health services by a volunteer peer for up to 6 months. A secondary aim of this study is, therefore, to investigate whether individual companionship increased participation in local communities, etc. through a self-assessed questionnaire given to the participants in the intervention arm. The primary hypothesis is that participants allocated to the PEER intervention added to SAU gain a significantly increased experience of self-assessed personal recovery compared to participants who are allocated to SAU alone. Additionally, we hypothesize that the superiority of the PEER intervention will be applicable for secondary outcomes and exploratory measures at post-intervention so that improvement in empowerment, hope, self-efficacy, self-advocacy, social network, quality of life, and work and social functioning will be significantly increased among participants allocated to the PEER intervention.

### Trial design {8}

The PEER trial is designed as a randomized, two-arm, investigator-initiated, multi-municipal, parallel-group superiority trial. The primary outcome is self-assessed personal recovery at end of the intervention. Secondary outcomes include self-assessed empowerment, quality of life, and work and social functioning. The PEER trial is reported in this article according to the Standard Protocol Items: Recommendations for Interventional Trials (SPIRIT) 2013 statement [11], and the final results will be published according to the Consolidated Standards of Reporting Trials (CONSORT) criteria for randomized trials of nonpharmacological treatment [12].

## Methods: Participants, interventions, and outcomes

### Study setting {9}

The intervention will be delivered by volunteer peers and in case of absence by local coordinators that are organized in collaboration with the non-governmental Peer Partnership organisation in the participating municipalities situated at five study sites in the municipalities of Elsinore, Greater Copenhagen, and Fredericia. Potential participants are informed about their opportunity to participate by local coordinators at introductory meetings about the group course and the PEER trial and by social workers from the municipal social services. Additionally, potential participants with similar mental health challenges can self-refer to the study. The 10-week group course (*N*_max_=10) is delivered by two volunteer peers aged 18 years or older with their own experiential skills with vulnerability to mental health difficulties and the group course is held in civil society locations other than the municipal social service and mental health centers. The volunteer peers and the local coordinators must complete a specific peer education to facilitate the PEER group course. Peers entering individual companionship must complete additional specific peer training.

### Eligibility criteria {10}

Eligible participants in this trial meet the following inclusion criteria:

1. Individuals using the municipal social service in the participating municipalities for support and assistance due to vulnerability to mental health difficulties, corresponding to the target group for §82 in the Danish law of social service—i.e., individuals diagnosed with a mental illness and/or who is affected by mental dissatisfaction to a degree that limits the unfolding of their life. Additionally, individuals who self-refer to the trial with similar mental health challenges.
2. Are residents of the participating municipalities at baseline.
3. Can understand, speak, and read Danish.
4. Are aged 18 years or older.
5. Have given verbal and written consent to participate in the trial.

Eligibility is assessed by the social workers and local coordinators in each municipality. The PEER intervention is not designed to accommodate individuals in need of acute or highly specialized care. Thus, potential participants will not be eligible if they meet the following criteria:

1. Individuals with alcohol and/or drug abuse are welcome in the group. However, if they according to the local coordinator’s judgment cannot participate in the peer group, they are advised to contact professional help.
2. Individuals with suicide thoughts are welcome in the group. However, if they have specific suicide plans and according to the local coordinator’s judgment cannot participate in the peer group, they are advised to contact professional help.

### Who will take informed consent? {26a}

The individual information interview and obtaining of informed consent is handled by the local coordinators, who is employed by the Peer Partnership association to take care of the individual contact with participants and the execution of the PEER intervention. Alternatively, the individual information will be handled by researchers employed at Copenhagen Research Center for Mental Health (CORE). The local coordinators and the researchers are thoroughly trained in the project protocol and in conducting individual information interviews. The individual information will be given either in continuation of the introductory meeting or at a subsequent individual meeting. It will be ensured that the individual conversation can take place calmly and undisturbed and that it is possible for potential participants to be assisted by a person of their own choice. The individual information contains adequate information about the PEER trial and the participants are informed that participation in the trial is voluntary, that the intervention is not estimated to have any adverse effects, and that they can withdraw from the trial at any time, without consequence for future municipal social and/or mental health service. If the potential participant, after being informed of the trial, remains interested in participating, the participant can be included in the trial, and an informed consent form must be signed. Participants are entitled to at least 24 hours of reflection time or more if needed.

### Additional consent provisions for collection and use of participant data and biological specimens {26b}

Not applicable

## Interventions

### Explanation for the choice of comparators {6b}

We aim to investigate if the PEER intervention added to SAU is superior to SAU. Therefore, we chose to compare the PEER intervention to SAU alone.

### Intervention description {11a}

#### The intervention

The development of the group intervention

The PEER intervention is a new community-based person-centered recovery-oriented intervention developed in co-creation by the Peer Partnership association and CORE, Recovery & Inclusion, Mental Health Center Copenhagen. The PEER intervention is inspired by: Peer support groups in the MIND organization in the UK [13]; Manuals for peer support services [14] and peer training [15], which has shown a positive effect on measures of personal recovery in RCTs; Practical guides to everyday life developed by consumers of mental health treatment in Denmark; and lived experiences of mental illness and recovery within the project group.

The PEER intervention is based on a theoretical model of change mechanisms for peer worker interventions by Gillard et al. [16] adapted to a community setting, as well as literature about collective action and co-production in public services [17, 18]. Key change mechanisms according to Gillard et al. were (1) building trusting relationships based on shared lived experience, (2) role-modeling individual recovery and living well with mental health problems, and (3) engaging service users with mental health services and the community [16]. The content of the group sessions is developed from themes identified in the CHIME (Connectedness; Hope; Identity; Meaning; Empowerment) framework as promoting the personal recovery process [19], as well as knowledge from systematic reviews and meta-analyses in the field focusing on the effect of peer support [5, 6, 9]. Additionally, the method of life storytelling [20] and the mindset of acceptance and commitment therapy (ACT) [21] have formed the basis of specific group sessions. The content and the form of specific group sessions were tested in three group courses (*N*=19 recipients of peer support; *N*=2 providers of peer support) conducted during 2019. Furthermore, a manual version 1 for the PEER intervention has been tested in a pilot study (*N*=53 recipients of peer support; *N*=12 providers of peer support) of seven group courses during the period Feb.–July 2020 in three participating municipalities. The pilot study was interrupted in March 2020 due to a national lockdown caused by the COVID-19 pandemic, resulting in participant dropout. Nevertheless, the groups were restarted in May 2020 in accordance with Danish health authority guidelines. The content of the manual has been further developed and revised through a qualitative pilot evaluation based on focus groups and telephone-based interviews with 16 recipients and nine providers of peer support participating in the pilot study. The qualitative evaluation clarified the specific purpose and aims of the intervention, which resulted in revision of the content of the intervention manual and the peer training guide. The final PEER intervention consists of a 10-week group course delivered by two volunteer peers and if needed the opportunity of individual companionship with a volunteer peer for up to 6 months. It is mandatory for the participants to participate in an introductory meeting with the purpose of being informed about the peer group, the individual companionship, and the RCT so that participation in the intervention becomes the participants' own informed choice. The entire PEER intervention is described in a comprehensive manual version 2 and detailed instructions have been prepared for the volunteer peers to make it accessible and ensure similarity across the groups. The overall content of the PEER intervention is presented in Table 1.

**Table 1:** The overall content of the group sessions in the “Paths to EvERyday life” (PEER) intervention

Themes in the manual	Overall content of the group sessions
#1: Arrival and getting started	The program is guided firmly by the peer group facilitators, making sure the group has a good start. The group comes to an agreement about what it takes for everyone to benefit from the group sessions.
#2: What to tell others, when...	The focus in the group is on individual boundaries and what we want to share about ourselves with others.
#3: Find out what's most important	Standing strong in everyday life. The focus in the group is on life values that matter for the individual.
#4: Similarities and differences	The focus is on differences and similarities in the group. Differences are a strength.
#5: Chance’s worth taking	The focus in the group is on chances worth taking, readiness for change, and individual safety.
#6: Revival of taking chances and our network	The peer group facilitators revive last week’s theme about chances and the focus is on what is needed to socialize with others and how interpersonal relationships enrich daily living.
#7: Life stories and narratives	The focus is on life storytelling in smaller groups as a key to control everyday life, to listen and be listened to.
#8: Setting the scene	The focus in the group is on practicing how to invite other people to help and support - to achieve what’s important for the individual.
#9: From dreaming to doing	The focus in the group is on making individual plans.
#10: Treat yourself!	The group exchanges experiences, tips, and tricks for balancing individual energy levels and cap off the group sessions in a meaningful way.

The overall purpose of the PEER intervention is to find a way to live life in a meaningful way – despite still finding some things challenging in everyday life. The aim is to form a constructive community through group sessions where exchanges of lived experiences, mutuality, and social network can develop. We hypothesize, that the volunteer peers by sharing their experiences with mental vulnerability and recovery can create trust and inspire the participants to safely share their own experiences. Additionally, that the volunteer peers by presenting group themes and by participating on an equal footing with the participants in the group exercises can contribute to the participants’ experience of connectedness with others, as well as promote the participants’ self-esteem and belief in possibilities, dreams, and aspirations to regain meaning in life circumstances, control and responsibility for their own life.

##### The individual companionship

Paths to EvERyday life (PEER) is developed with two intervention components. If needed participants allocated to the PEER group intervention have the opportunity of individual companionship with a volunteer peer for up to 6 months after the participants is allocated to the group course. The purpose of the companionship is to reduce the barrier for the participants to participate in activities that are important for individual well-being and participation in everyday life, including participation in volunteer work, leisure activities, education, meetings with authorities, etc. We hypothesize, that by joining communities in the local area, peer support can support social inclusion and participation. The empowerment-promoting dimension of the peer support is hypothesized to contribute to the citizens’ ability to make wishes and needs clear and understandable to the authority. It is the local coordinator who, through dialogue, assesses the need for companionship and matches the participants with a volunteer peer. The volunteer peers help to clarify the participants’ expectations and motivation and provide companionship. The volunteer peers offer companionship as a personal support and will not act as an assessor or responsible party. In the pilot study, the individual companionship was not fully implemented due to a lack of time to prioritize the companionship on an equal footing with the implementation of the group courses, and some of the participants already received companionship from e.g., the municipality. In addition, national restrictions related to reducing the spread of COVID-19 might have had an impact on access to local society activities, etc.

##### Evaluation on individual, systemic, and society level

The PEER trial is part of an overall project with the aim of evaluating the PEER intervention at individual, systemic, and society level. Initially, we aimed to investigate the effect of the PEER intervention both at post-intervention and at 6-month follow-up. However, we only received funding for the 3-month follow-up. Therefore, we decided to apply for a feasibility study of the individual companionship instead of the 6-month follow-up. However, we did not get the funding. Nevertheless, we decided to investigate whether the PEER intervention has an impact on access to local communities, etc. through a 6-month follow-up questionnaire given to the participants in the intervention arm. As part of the overall project, the individual experiences of the recipients and the providers of the PEER intervention will be investigated with qualitative methods in a process evaluation of this RCT. Furthermore, whether the PEER intervention has an impact on systemic changes will be evaluated with Outcome Harvesting methods. Lastly, the cost-effectiveness of the PEER intervention will be evaluated.

##### Training and supervision

The volunteer peers have their own experiences with vulnerability to mental health difficulties and are in touch with their own recovery, i.e., they will be further along in their personal recovery process and will have the competencies and experiences to support the participants to find their own paths in everyday life [22]. The volunteer peers can work both as a group facilitator and individual companion. Volunteers must complete a specific peer education that will prepare them to facilitate PEER group sessions and enter individual companionship. The education consists of a training weekend, as well as written material with background information about: The PEER intervention and the RCT; The social rules of group interaction; How adults learn; How to use the language as experiential competence; and Guidance in facilitating trauma-informed peer support [23]. In addition, the focus of the training is on how to get a group process going and how the volunteer peers can handle difficult situations and, e.g., refer to other counseling services. Volunteer peers can receive professional sparring and supervision during the project and are provided with a comprehensive manual, which contains both general instructions and detailed instructions for each group session. The training can, if necessary, be conducted as online teaching.

#### Service as usual (SAU)

All participants in the trial will receive SAU by their social worker, or no specific service if the participant has been allocated to the trial by self-referral. Participants who are referred to the trial via §82 in the municipality, can receive other §82 offers depending on the individual municipality. In the municipality of Fredericia, adult citizens with mental health difficulties are able to enter directly from the street in “Your Entrance” and receive individual goal-oriented counseling and group-based offers by social workers under the themes of emotion/life management, mental vulnerability, ear acupuncture and socializing. Additionally, “Your Entrance” offer individual companionship with a volunteer to activities in the local environment. Citizens in Fredericia can receive support for up to 5 months. In the municipality of Copenhagen, adult citizens with mental health difficulties can participate in three intro workshops under §82 focusing on inspiring personal recovery, connectedness, and having an active life with interests. Furthermore, it is possible to receive individual support from a contact person educated as a social worker and/or with their own experiences with mental vulnerability. The purpose is to strengthen the decision-making skills of the individual citizen and to offer individual companionship for activities in the local environment. Citizens in Copenhagen can receive support from the contact person for up to 6 months. In Elsinore and the surrounding municipalities of Copenhagen, the introduction of §82 offers is in the preliminary phase.

### Criteria for discontinuing or modifying allocated interventions {11b}

Not applicable

### Strategies to improve adherence to interventions {11c}

The purpose of the introductory meeting is to inform about the content of the intervention and the trial. Thus, participation in the PEER trial is the participants’ own informed choice. Moreover, the participants are informed that attendance in the group sessions is important to ensure group dynamics. To enhance attendance, a text message is sent as a reminder on the same day as group sessions are held. In cases of absence from the group, the participants are encouraged to send a text to the group. If needed, participants can contact the local coordinators between group sessions. A fidelity scale is developed and used for biannual fidelity reviews to ensure intervention program adherence and continuous focus on program implementation and improvement. Once program fidelity is achieved, future fidelity reviews will be conducted annually.

### Relevant concomitant care permitted or prohibited during the trial {11d}

All participants in the trial can also receive other forms of support, such as coping services, psychotherapy and outpatient psychiatric treatment or any other offers of the participants’ own choice.

### Provisions for post-trial care {30}

Not applicable

### Outcomes {12}

The primary, secondary, and exploratory outcomes are presented in Table 3.

#### Primary outcome

- Personal recovery—measured by Questionnaire about Process of Recovery (QPR-15) [24, 25] at the end of intervention (3 months after allocation).

#### Secondary outcomes

- Empowerment—measured at the Empowerment scale [26] at the end of intervention (3 months after allocation).
- Work and social function—measured by the Work and Social Adjustment Scale (WSAS) [27] at the end of intervention (3 months after allocation).
- Quality of life—measured by the Manchester Short Assessment of Quality of Life (MANSA) [28] at the end of intervention (3 months after allocation).

#### Exploratory outcomes

- Self-efficacy is measured by the General Self-Efficiency Scale (GSE) [29] after completion of intervention at the end of intervention (3 months after allocation).
- Hope—measured at the State Hope Scale [30] at the end of intervention (3 months after allocation).
- Self-advocacy—measured at the Self-Advocacy Scale (SAS) [31] at the end of intervention (3 months after allocation).
- Social network—measured by the Copenhagen Social Relations Questionnaire (CSRQ) [32] at the end of intervention (3 months after allocation).

#### Safety measures

The PEER intervention is not expected to have any adverse effects. However, if anyone experiences distress during the groups through either unrelated or related to the intervention the participants can reach out to either the group facilitators and/or local coordinators. For example, if someone discloses a serious risk of harm during the group, the participants will be informed about other relevant counseling services and/or encouraged to contact professional help. When trial recruitment and the intervention phase have ended, safety measures, i.e., the number of somatic and psychiatric hospitalization days, death, suicide, and probable self-harm are obtained from the Danish central registers to examine any unexpected adverse effects during the intervention period (Table 3).

### Participant timeline {13}

**Fig. 1:**
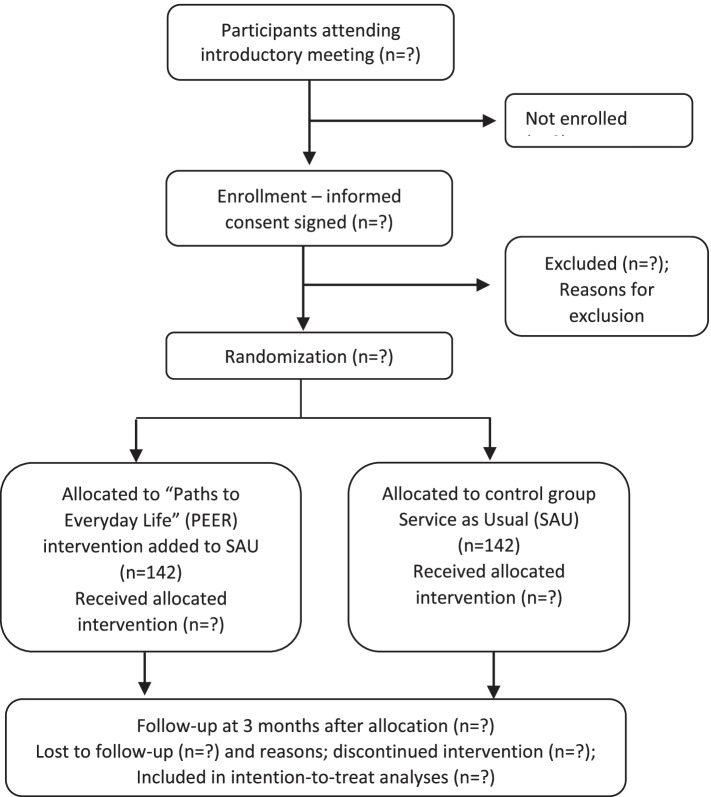
Flowchart of participant timeline

**Fig. 2:**
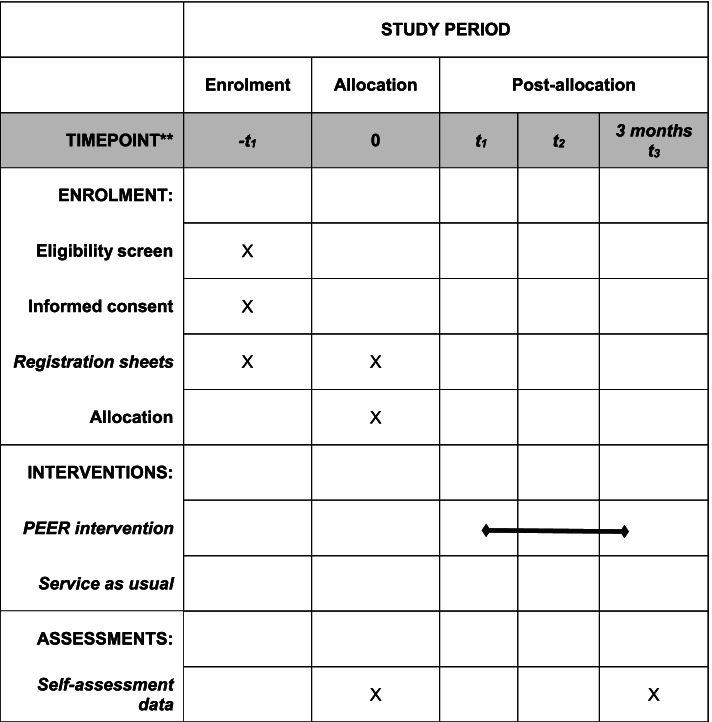
Schedule of enrolment, interventions, and assessments

Partipant timeline is presented in Fig. 1. The time schedule of enrolment, interventions, and assessments for participants is presented in Fig. 2.

### Sample size {14}

The sample size calculation is based on tracking a minimal but clinically significant difference between the intervention group and the control group on the continuous scale QPR-15. The RCT by Johnson et al. (2018) recruited a broad target group of individuals who approached psychiatric crisis centers. The response to QPR-22 was normally distributed and with a standard deviation (SD) of 16 in the intervention and control group at end of intervention at 4 months [15]. In the present RCT, we plan to measure QPR-15 at end of intervention at 3 months and therefore choose a SD of 15 based on the hypothesis that the SD increases over time. A minimum clinically relevant difference between the intervention and control group between 4-5 points is recommended [25, 33]. With an allocation ratio of 1:1 and a minimum clinically relevant difference of 5, a power of 80%, and a significance level of 0.05%, we need 284 participants, i.e., 142 in the intervention group and 142 in the control group (Fig. 1) to reject the null hypothesis that self-assessed personal recovery is equal in the control group and the PEER group. The sample size and power calculations are conducted using PS Power and Sample Size Calculations software [34]. Power calculations (Table 2) indicate that a sample size of 142 participants per group will be adequate to detect relevant significant differences in the secondary outcome measures with a minimum power of 80%.

**Table 2:** Power calculations for secondary outcomes

Outcome	δ-value for the clinically relevant difference in means	σ-value for expected SD	α	Power	Test	Reference
Empowerment Scale, Rogers (ESR)	0.2	0.35	0.05	0.998	*t* test	[26, 35]
Manchester Short Assessment of Quality of life (MANSA)	6	1	0.05	1.0	*t* test	[28, 36]
The Work and Social adjustment scale (WSAS)	4	10	0.05	0.920	*t* test	[27, 37]

### Recruitment {15}

The staff at the municipal social services inform potential participants about the project and that they can get further information by contacting the local coordinator themselves and/or participating in the introductory meeting. Additionally, the PEER intervention is disseminated via information meetings in the municipality (e.g., §82 offers, job centers, and social psychiatry), as well as collaboration agreements with local contacts (e.g., SIND – national association for mental health and Headspace). In addition, flyers are distributed, and posters are hung up in shops, cafés, libraries, universities, and community houses. The project is also advertised online via the Peer Partnership’s website, social media, and in press. Furthermore, first contacts can be made by potential participants contacting the local coordinator or attending the introductory meeting after becoming aware of the project via an announcement. In all cases, the participants must contact the local coordinator themselves and/or participate in the introductory meeting. No further contact is made with potential participants unless they contact the project on their own initiative. Strategies for achieving adequate participant enrolment to reach the target sample size are regular meetings with both leaders in the municipalities, as well as social workers likely to be in contact with the target group. Presentations about the project and street events, as well as increasing public awareness through social media.

## Assignment of interventions: allocation

### Sequence generation {16a}

The REDCap (Research Electronic Data Capture) randomization tool will be used to facilitate randomization. REDCap is an electronic data capture tool hosted at the Capital Region of Denmark. REDCap is a secure, web-based software platform designed to support data capture for research studies [38, 39]. Access to data requires a two-step unique log-in system and is granted to researchers and staff affiliated with the project. A trial audit will not be conducted due to a lack of funding. The allocation ratio between the two arms is 1:1. The allocation sequence will be stratified by the municipality study site.

### Concealment mechanism {16b}

To ensure concealment, the randomization schedule is stored away from the research team and the block sizes are not disclosed. The allocation is performed by a not-blinded research coordinator, who informs the participants allocated to the control group through a central telephone. Moreover, the research coordinator informs the local coordinators in each municipality about participants allocated to the intervention group through submitting the record id via secure email. The local coordinators will be able to identify the participants through their access to REDCap.

### Implementation {16c}

A researcher not involved in the trial will create the randomization allocation tables that will be uploaded to REDCap. The randomization allocation tables will be generated according to study design specifications as determined by the researcher and investigator/s. Once local coordinators or researchers have enrolled the participants, the researchers send baseline questionnaires to the participant, either electronically or exceptionally by letter. When the baseline questionnaire has been answered, participants will be randomized when the un-blinded research coordinator enters a participant’s REDCap record and click the “Randomize” button. Clicking this button triggers REDCap to check the allocation table and display the group to which the participant should be randomly assigned. This assignment is permanent and not editable within the participant record and, like all other activity within REDCap, is tracked and not modifiable in the audit log.

Participants who are randomized to the control group are informed by the research coordinator. Participants allocated to the PEER intervention are informed by the local coordinator who will assign participants to start a 10-week group course.

## Assignment of interventions: Blinding

### Who will be blinded {17a}

Due to the nature of the PEER intervention, the participants, the local coordinators, and the volunteer peers delivering the intervention cannot be blinded to the group allocation. All outcomes are based on self-assessed questionnaire data, and no assessor-based follow-up data will be obtained. Register data on safety measurements, i.e., hospitalization, death, and suicide attempts are created automatically through the national registries. Information on the participant’s attendance in the group sessions is registered through the REDCap software system by the local coordinators. The researchers will be blinded to group allocation during the process of data management and data analysis. Group allocation will be coded with names like X and Y to conceal the given intervention. The researcher will draw up conclusions at post-intervention based on scenarios where each group (X and Y) has received the PEER intervention and added SAU and SAU alone.

### Procedure for unblinding if needed {17b}

Not applicable

## Data collection and management

### Plans for assessment and collection of outcomes {18a}

The participants will be followed up at end of the intervention. The questionnaire contains primary, secondary, and exploratory outcomes, which is presented in Table 3. The primary outcome is self-assessed personal recovery from baseline to end of intervention (3 months after allocation). Personal recovery is measured with the Questionnaire about the Process of Recovery (QPR), which consist of 15 items measuring aspects of personal recovery – based on mental health consumer experiences of recovery [40]. In a systematic review, QPR has been found to have the closest match with the CHIME (Connectedness; Hope; Identity; Meaning; Empowerment) framework compared to other recovery measures in the field [41]. In psychometric evaluations, QPR-15 demonstrated good internal consistency and test-retest reliability, as well as sufficient convergent validity and moderate sensitivity to change [24, 25]. Each item is scored on a 5-point Likert scale ranging from 0 (strongly disagree) to 5 (strongly agree) and gives a total score between 0-60. Secondary outcomes include self-assessed empowerment, quality of life, and functioning. Empowerment is measured with The Empowerment scale Rogers (ESR), which consist of 28 items measuring a person’s resources, opportunities, and sense of control over their own life – based on mental health consumer experiences of empowerment [26]. The Empowerment scale is widely used and validated and scored on a 4-point Likert scale ranging from 0 (strongly disagree) to 4 (strongly agree) [42]. Quality of life is measured with The Manchester Short Assessment of Quality of life (MANSA), which consist of 16 items where 4 items measure objective quality of life (close relationships, contact with friends, crime, and assault) and 12 items measure subjective quality of life (satisfaction with life as a whole, work, financial situation, friendships, leisure activities, housing, personal safety/security, cohabitation, sex life, family relationships, and health). The questionnaire has been validated [28, 43] and is scored on a 7-point scale ranging from 1 (couldn’t be worse) to 7 (couldn’t be better). Functioning is measured with The Work and Social Adjustment Scale (WSAS), which is a 5-item self-assessed questionnaire covering a person’s perceived functioning in terms of the domains (1) workability, (2) performing tasks at home (cleaning, shopping, paying bills, etc.), (3) social leisure activities (parties, dating, tours, visits, cinema, etc.), (4) private leisure activities (reading, gardening, sewing, walking alone, etc.), and (5) ability to form and maintain close relationships. The questionnaire is widely used and validated [44] and scored on an 8-point scale ranging from 0 (not at all) to 8 (very seriously). Explorative outcomes included self-efficacy, hope, self-advocacy, and social network. Self-efficacy is measured with the General Self-efficacy (GSE) scale, which consists of 10 items designed to assess optimistic self-beliefs to cope with a variety of difficult demands in life. The GSE scale is widely used and validated [29, 45] and is scored on a 4-point Likert scale ranging from 0 (not at all true) to 4 (exactly true). Hope is measured with the State Hope Scale (SHS) scale, which consist of 6-items measuring hope, i.e., the belief in one’s own ability to initiate and maintain actions and ways to achieve goals. The SHS scale is widely used and validated [30] and scored on an 8-point scale ranging from 1 (definitely false) to 8 (definitely true). Self-advocacy is measured with the Self-Advocacy Scale (SAS), which consists of 8 items involving taking care of yourself, being organized and prepared, finding the resources you need, and communicating and negotiating to get your needs met. The SAS scale is only used and validated in research about acquired brain injury [31] and scored on a 4-point Likert scale ranging from 0 (not confident) to 4 (very confident). Social network is measured with a modified version of the Copenhagen Social Relations Questionnaire (CSRQ), which consist of 19 items covering frequency of social contact, social support in everyday life, quality of social relations, and frequency of participating in local social activities. The questionnaire has satisfactory validity and reliability and is widely used in Danish population surveys [32].

**Table 3:** Primary, secondary, and exploratory outcomes, safety measures, and data collection

	Data source	Outcome	Baseline	3 months	At the end of the trial
Primary	Questionnaire	Difference in personal recovery measured by Questionnaire about Process of Recovery (QPR-15)	X	X	
Secondary	Questionnaire	Difference in empowerment measured by The Empowerment Scale, Rogers (ESR)	X	X	
	Questionnaire	Difference in quality of life measured with The Manchester Short Assessment of Quality of life (MANSA)	X	X	
	Questionnaire	Difference in functioning measured with The Work and Social Adjusment Scale (WSAS)	X	X	
Exploratory	Questionnaire	Difference in self-efficacy measured with the General Self-efficacy (GSE) scale	X	X	
	Questionnaire	Difference in hope measured with the State Hope Scale (SHS)	X	X	
	Questionnaire	Difference in self-advocacy measured with the Self-Advocacy Scale (SAS)	X	X	
	Questionnaire	Difference in social network measured with a modified version of the Copenhagen Social Relations Questionnaire (CSRQ)	X	X	
Safety	National Patient Register	Number of hospitalization days			X
	Register of causes of death	Death			
	National Patient Register; Psychiatric Central Research Register	Suicide and probable self-harm			X

### Plans to promote participant retention and complete follow-up {18b}

The participants are reminded or prompted to complete the questionnaires through personal contact (text or phone call). To enhance adherence to the trial, participants are assisted if they wish by a research assistant at follow-up and the follow-up schedule is as flexible as possible to minimize the burden on the participants. A recoding of record id will be performed in the data to ensure that the research assistant remains blinded to group allocation during the questionnaire interviews. The participants are informed not to disclose their group allocation. If group allocation is disclosed during the interview, another research assistant will take over the interview. If participants want to discontinue the intervention, there are three options:

1. They discontinue the intervention, but not the experiment, i.e., they would like to answer questionnaires after the end of the intervention.
2. They discontinue both the intervention and the experiment, but data can be retained and used in the research project.
3. They discontinue both the intervention and the experiment and data cannot be used and must be deleted.

### Data management {19}

The schedule of enrolment, interventions, and assessments is shown in Fig. 2. Number of potential participants attending the introductory meetings is registered by the local coordinators. Informed consent is registered in REDCap. The researchers send baseline questionnaires to the participant, either electronically, or exceptionally by letter. Instructions to participants about responding to the questionnaire are included in the questionnaire. If needed, the participant can respond to the questionnaires with assistance from a researcher through a central telephone. Attendance in PEER group sessions is registered in the intervention arm by the local coordinators in REDCap. Follow-up data are obtained using the same questionnaires as used at baseline. Questionnaires are sent to all participants, including those who have decided to withdraw from the intervention, unless they have withdrawn their consent to participate in the study. Study data are collected and stored using REDCap. Data quality will be promoted through monthly data monitoring conducted by the research coordinator for data completeness and verification of data, e.g., personal identification number. Missing or invalid data are reported directly to the researchers. Secondly, REDCap has functions designed to detect missing data and certain errors in data, e.g., invalid values. Lastly, final data control will be performed before data analysis by the research coordinator. Participant files are stored for up to 10 years after study completion.

### Confidentiality {27}

All data will be stored in accordance with the European General Data Protection Regulation, as well as national guidelines. Personal information on participants is entered directly into REDCap. Only data on participants that have consented to participation in the trial will be stored. At the termination of the trial, data will be transferred to the national archives in accordance with Danish legislation.

### Plans for collection, laboratory evaluation, and storage of biological specimens for genetic or molecular analysis in this trial/future use {33}

Not applicable

## Statistical methods

### Statistical methods for primary and secondary outcomes {20a}

The primary outcome is personal recovery measured on QPR-15 at post-intervention (3 months). Primary and secondary outcomes are continuous. Differences between the intervention group and the control group will be analyzed using analysis of covariance (ANCOVA). ANCOVA is a general linear model that tests whether the average of a dependent variable is similar across levels of a categorically independent variable, in this case, the PEER intervention, while statistically controlling for the effects of other continuous variables, i.e., co-variates that are not of primary interest. An elaborate analysis plan will be prepared before the analytic phase and will be uploaded to clinicaltrials.gov.

### Interim analyses {21b}

Not applicable

### Methods for additional analyses (e.g., subgroup analyses) {20b}

An elaborate analysis plan will be prepared before the analytic phase and will be uploaded to clinicaltrials.gov.

### Methods in analysis to handle protocol non-adherence and any statistical methods to handle missing data {20c}

Data analyses will be based on the intention-to-treat principle, i.e., that data from all participants will be included corresponding to the group to which the participants have been allocated. In case of missing data, multiple multivariate imputations will be used and all co-variates of supposed prognostic significance will be used to impute a distribution of missing data.

### Plans to give access to the full protocol, participant-level data and statistical code {31c}

We give access to the full protocol via Clinicaltrials.gov. Access to participant-level data is not applicable due to Danish data protection law.

## Oversight and monitoring

### Composition of the coordinating center and trial steering committee {5d}

The Peer Partnership association manages the operational part of the trial, i.e., the collaboration with the local coordinators in each municipality, training, and supervision of the volunteer peers delivering the PEER intervention. The steering group of the project keeps track of the progress and decisions regarding the scientific content of the project. The Peer Partnership association and the steering group will not take part in decisions regarding data analysis, the interpretation, or the publication of results.

### Composition of the data monitoring committee, its role, and reporting structure {21a}

Not applicable

### Adverse event reporting and harms {22}

The PEER intervention is not estimated to have any adverse effects or harms. However, it is expected that the intervention targeting the individual recovery process can initiate a distressing journey. The groups talk openly about this and the participants are encouraged to reach out to group facilitators who will reach out to the local coordinators if needed. The local coordinators and the volunteer peers have been instructed to inform participants in crisis about regional and local counseling services. It is also considered that facilitating the group courses can put a strain on the volunteer peers. Therefore, the volunteer peers are offered professional sparring with local coordinators and supervision locally in the municipalities and are informed to be aware and open about their own warning signs. The local coordinators are trained to step in as a substitute for the volunteer peers in the event of illness and absence.

### Frequency and plans for auditing trial conduct {23}

Not applicable

### Plans for communicating important protocol amendments to relevant parties (e.g., trial participants, ethical committees) {25}

Important protocol modifications will be notified to the relevant parties, e.g., the regional ethics committees of the Capital Region, the Data protection agency, Clinicaltrials.gov, the steering group, the Peer Partnership association, and the trial participants.

### Dissemination plans {31a}

The project is disseminated locally and nationally. The results of the project will be presented at relevant international conferences. In all dissemination activities, the role of the VELUX FOUNDATION as a donor will be included, and the project will be available for the dissemination activities of the VELUX FOUNDATION. In addition, at least two original articles with the results of the evaluation will be published in international scientific journals. Positive as well as negative and neutral results will be published. Authorship is determined by the Vancouver criteria.

## Discussion

This paper describes the study protocol of a randomized controlled trial comparing (1) PEER intervention added to SAU, with (2) SAU for individuals with vulnerability to mental health difficulties. Mental vulnerability is a frequent cause of impaired personal recovery and mental health, with great costs to the individual and society. New innovative interventions are necessary to prevent people from developing severe mental illnesses. The PEER trial will test a new community-based peer-support intervention, and if the intervention proves to be effective, the goal is that future integration of the PEER intervention will improve individual recovery and mental health and reduce the societal burden of individuals seeking municipal social support and/or mental health services.

This randomized controlled trial is designed with great emphasis on minimizing bias, and reporting is done in accordance with SPIRIT guidelines [11]. This study has several methodological strengths, including (1) the sample size is large, and hence we expect high statistical power, which allows for detection of relevant differences in both primary and secondary outcomes; (2) the randomization is done in accordance with high methodological standards; (3) the primary outcome is based on a validated self-assessed questionnaire covering all five domains of personal recovery in the CHIME framework [19, 41]; and (4) the PEER intervention has been tested and qualitatively evaluated in a prior pilot study of seven group courses with 25 respondents in the three participating municipalities.

Since the nature of the intervention does not permit blinding of the participants, local coordinators, or volunteer peers, it may increase the risk of expectancy and performance bias. Thus, it is a possibility that it may have an impact on the participants’ responses to the self-assessed questionnaires. We are attempting to minimize this bias by striving to obtain answers to questionnaires from all participants allocated to the trial including those discontinuing the intervention. Also, multiple multivariate imputations will be used in case of missing data. Moreover, the limitations include that the target group is expected to be broad due to a broad recruitment strategy and the possibility to self-refer to the trial. Moreover, SAU for persons with vulnerability to mental health difficulties is scarcely described in Denmark. Thus, a limitation in the study design is the limited knowledge about the quality and quantity of the control intervention. We expect possible differences in effects between the participating municipalities because of variations in the standard municipal social §82 services across the municipalities. We are attempting to minimize this bias by stratifying the randomization for municipality. Additionally, fidelity reviews will be conducted to explicate differences in implementation of the PEER intervention. Lastly, we expect that facilitating the group sessions can put a strain on the volunteer peers with the risk of illness and absence. We address this by offering the volunteer peers professional supervision with local coordinators, and locally in the municipalities. Additionally, the volunteer peers are informed to be aware and open about their own warning signs. The local coordinators are trained to act as a peer substitute in cases of illness and absence. Nevertheless, the limitations of the study must be considered when interpreting the findings.

This study can contribute to new knowledge on community-based peer support interventions in welfare societies. If this trial shows that the PEER intervention is superior to SAU, these positive results will support the further development of enhanced community-based municipal social service for persons with vulnerability to mental health difficulties, and a wider implementation of the PEER intervention can be recommended.

## Trial status

The PEER trial started recruiting in December 2020. This protocol is version 4.0. Trial recruitment is expected to end in Oct. 2022.

## Data Availability

Not applicable

## Abbreviations

ACT: Acceptance and Commitment Therapy
ANCOVA: Analysis of Covariance
CHIME: Connectedness; Hope; Identity; Meaning; Empowerment
CONSORT: Consolidated Standards of Reporting Trials
CORE: Copenhagen Research Center for Mental Health
CRF: Case report form
CSRQ: Copenhagen Social Relations Questionnaire
ESR: The Empowerment Scale Rogers
GSE: General Self-Efficacy Scale
ICD-10: International Classification of diseases version 10
ImROC: Implementing Recovery through Organizational Change
MANSA: Manchester Short Assessment of Quality of life
PEER: Paths to EvERyday life
QPR: Questionnaire about the Process of Recovery
RCT: Randomized controlled trial
REDCap: Research Electronic Data Capture
SAS: The Self-Advocacy Scale
SHS: State Hope Scale
SPIRIT: Standard Protocol Items: Recommendations for Interventional Trials
WSAS: Work and Social Adjustment Scale
